# Hospital-based prospective study of pertussis in infants and close contacts in Tehran, Iran

**DOI:** 10.1186/s12879-021-06266-6

**Published:** 2021-06-18

**Authors:** Gaelle Noel, Masoumeh Nakhost Lotfi, Sajedeh Mirshahvalad, Sedaghatpour Mahdi, David Tavel, Seyed M. Zahraei, Roxana Mansour Ghanaie, Tahereh Heidary, Aliahmad Goudarzi, Azardokht Kazemi, Abdollah Karimi, Alireza Nateghian, Mohand Ait-Ahmed, Nicole Guiso, Fereshteh Shahcheraghi, Fabien Taieb

**Affiliations:** 1 Center for Translational Science, Institut Pasteur, Paris, France; 2 Department of Bacteriology, Pertussis Reference Laboratory, Pasteur Institute of Iran, Tehran, Islamic Republic of Iran; 3 Emerging Diseases Epidemiology unit, Institut Pasteur, Paris, France; 4 Center for Communicable Diseases Control, Ministry of Health and Medical Education, Tehran, Islamic Republic of Iran; 5 Pediatric Infections Research Center, Mofid Children’s Hospital, Shahid Beheshti University of Medical Sciences, Research Institute for Children’s Health, Tehran, Islamic Republic of Iran; 6 Department of Pediatrics, Ali Asghar children hospital, Iran University of Medical Sciences, Tehran, Islamic Republic of Iran; 7 Pediatric cardiology department, Mofid Children’s Hospital, Shahid Beheshti University of Medical Sciences, Tehran, Islamic Republic of Iran; 8 Emergency Department, Mofid Children’s Hospital, Shahid Beheshti University of Medical Sciences, Tehran, Islamic Republic of Iran; 9 Centre for Translational Science, Clinical Coordination, Institut Pasteur, Paris, France; 10 Department of International Affairs, Institut Pasteur, Paris, France

**Keywords:** Pertussis, Infant, Whole-cell vaccine, Vaccine compliance, Infection, Diagnosis, Contact

## Abstract

**Background:**

Pertussis remain a global health concern, especially in infants too young to initiate their vaccination. Effective vaccination and high coverage limit the circulation of the pathogen, yet duration of protection is limited and boosters are recommended during a lifetime. In Iran, boosters are given at 18 months and 6 years old using whole pertussis vaccines for which efficacy is not known, and pertussis surveillance is scant with only sporadic biological diagnosis. Burden of pertussis is not well understood and local data are needed.

**Methods:**

Hospital-based prospective study implementing molecular laboratory testing in infants aged ≤6 months and presenting ≥5 days of cough associated to one pertussis-like symptom in Tehran. Household and non-household contact cases of positive infants were evaluated by comprehensive pertussis diagnosis (molecular testing and serology) regardless of clinical signs. Clinical evaluation and source of infection were described.

**Results:**

A total of 247 infants and 130 contact cases were enrolled. Pertussis diagnosis result was obtained for 199 infants and 104 contact cases. Infant population was mostly < 3 months old (79.9%; 157/199) and unvaccinated (62.3%; 124/199), 20.1% (40/199) of them were confirmed having *B. pertussis* infection. Greater cough duration and lymphocyte counts were the only symptoms associated to positivity. Half of the contact cases (51.0%; 53/104) had a *B. pertussis* infection, median age was 31 years old. A proportion of 28.3% (15/53) positive contacts did not report any symptom. However, 67.9% (36/53) and 3.8% (2/53) of them reported cough at inclusion or during the study, including 20.8% (11/53) who started coughing ≥7 days before infant cough onset. Overall, only five samples were successfully cultured.

**Conclusion:**

These data evidenced the significant prevalence of pertussis infection among paucy or poorly symptomatic contacts of infants with pertussis infection. Widespread usage of molecular testing should be implemented to identify *B. pertussis* infections.

**Supplementary Information:**

The online version contains supplementary material available at 10.1186/s12879-021-06266-6.

## Introduction

Pertussis (whooping cough) is a highly contagious respiratory disease caused by *Bordetella pertussis* or, in a lower extent, *Bordetella parapertussis*, typically transmitted through airborne droplets. Vaccines have been developed since 1940s and are now used globally, including in Low and Middle-Income Countries (LMIC) since the inception Expanded Programme on Immunization in 1974. Mass vaccination led to an important decrease in pertussis incidence, however, the pathogen still circulates worldwide even in countries where vaccine coverage is high in infants and young children. It remains an important cause of morbidity and mortality among infants too young to have initiated their primary vaccine series, who account for the majority of pertussis related complications, hospitalizations and deaths. Yeung and colleagues estimated that around 24.1 million cases and 160,700 deaths from pertussis in children younger than 5 years occurred in 2014; 21% cases and 53% of deaths being attributed to children less than 1 year of age [1]. To limit the period of vulnerability for infants, the World Health Organization (WHO) recommends to initiate the pertussis vaccination at 6 weeks of age and no later than 8 weeks, and maintain high coverage (≥90%) with at least three doses of quality assured pertussis vaccine [2, 3].

Modeling studies showed that LMIC contribute for the largest share of pertussis cases and deaths, however accurate data are limited in those countries [4]. In addition, LMIC use whole-cell vaccines (wPVs) for which immunogenicity and efficacy have not been analyzed. The various wPVs manufactured in the past and largely studied in the 1990s in high-income countries (HIC), where acellular vaccines (aPVs) are now mostly used, exhibited efficacy variability, which raises the question of the contemporary wPV effectiveness. It is thus necessary to gather information on pertussis epidemiology in those countries.

The pertussis vaccination program in Iran includes a 3-dose primary vaccination at 2, 4 and 6 months of life, and two additional doses at 18 months and 5–6 years of life with wPVs mainly provided by the Serum Institute of India. Based on WHO and United Nations Children’s Fund (UNICEF) estimates, vaccine coverage ≥95% for 1st and 3rd dose is maintained since the early 1990s, when number of cases dropped down [5, 6]. Still, pertussis cases are reported every year and we observed an increasing notification since 2007 [6]. Pertussis Reference Laboratory in Tehran has been isolating clinical pertussis strains from samples from different part of the country but number of samples only represent a small part of the notified cases [7, 8]. Studies using most recent sensitive technology is limited but available data showed that, as observed elsewhere, infants younger than 1 year of age had the highest pertussis incidence in Iran, and the circulation of the pathogen has been documented among older children and adolescents [9–11]. Yet, burden of pertussis is unclear in Iran and clinical and biological surveillance remain scarce.

This study aimed at improving our knowledge of pertussis in infants with whooping cough syndrome in Tehran, Iran, and at better understanding the source of infection by analyzing the close contacts of infected infants.

## Methods

### Study population and design

This hospital-based prospective study was conducted in Tehran, Iran, from November 2016 to May 2019. Infants were recruited at the Mofid Children’s Hospital (site 1) and Hazrat-e-Ali Asghar Hospital (site 2). Male and female infants were eligible for enrollment as index case if they were being aged ≤6 months, either with a persistent cough for at least 5 days associated to at least one symptom among apnea, inspiratory “whooping”, or post coughing vomiting, or with a persistent cough and a confirmed case of whooping cough in the entourage. Upon enrollment of index case, two nasopharyngeal samples were collected, and data on age, sex, size and weight, birth information, pertussis vaccination history, type and duration of pertussis-related symptoms, biological test results, antibiotic treatment type and duration, previous consultation, family composition and case of cough in the entourage, animal contact, were collected. Follow-up on pertussis-related symptoms, antibiotic treatment, hospitalization and disease outcome was performed at day 7 and 14 after inclusion.

For each PCR-positive index case, up to five contact cases, living or not in the same household, were recruited within 14 days from respective index case inclusion. Inclusion criterion was having had a regular contact (> 1 h/day) with the index case for at least 5 days before symptom onset in the infant. Upon enrollment of contact case, two nasopharyngeal samples and one capillary blood sample were collected, and data on age, gender, relationship with index, pertussis vaccination history, type and duration of past and current pertussis-related symptoms, antibiotic treatment type and duration and previous consultation were collected. Follow-up on cough duration, antibiotic treatment and disease outcome was performed at day 7 and, in case of pertussis infection confirmation, day 14 after inclusion.

Data were collected in standardized paper questionnaires and recorded in a computerized database.

### Biological sample collection and analysis

All the sampling material and laboratory reagents were provided by Institut Pasteur, Paris, and shipped to the Institut Pasteur of Iran.

Samples were collected by nurses in a standardized manner across study sites. Two nasopharyngeal samples were collected for each participant using Eswab containing Amies transport medium (Dacron, Ref. 482 CE). In contact cases, one blood sample (200–400 μl) was also collected in SST microtainer tubes from the tip of a finger using a lancet 23G needle (Becton Dickinson, Ref. 365,968 and 369,523, respectively). Nasopharyngeal and serum samples were stored at 15–25 °C and sent within 6 h to the Bacteriology Laboratory at the Institut Pasteur of Iran in Tehran.

Quantity controls were blindly tested before initiating the study and the principle of the walking forward logic for PCR analysis was employed to ensure quality of the result.

#### Real-time quantitative polymerase chain reaction (qPCR) analysis and bacterial culture for direct diagnosis

Upon reception by the laboratory, nasopharyngeal samples were kept at 4 °C. One nasopharyngeal sample was used for qPCR analysis, the other one for culture.

For qPCR analysis, DNA extraction was performed within 48 h. When not possible, sample was stored at − 20 °C until processing. Nasopharyngeal sample was homogenized by vortex and swab was discarded. Total DNA was extracted from 100 μl of the transport media (pure sample) and from 10 μl of the transport media spiked into 90 μl of sterile water (diluted sample), using the commercial High Pure PCR Template preparation kit (Roche). For each sample, both pure and diluted DNA extract solutions were tested by qPCR to address possible PCR inhibition. qPCR were carried-out using the commercial LightCycler 480 Probes Master kit (Roche), forward and reverse primers 0.5 μM and Taqman probe 0.2 μM (TIB Molbiol), DMSO 2.5% (Sigma), and 5 μl of DNA extract solution. Amplification of insertion elements IS*481* (*Bordetella spp.*) and IS*1001* (*B. parapertussis)* was first assessed [12–14]. IS*481* qPCR is very sensitive but not specific as it targets DNA of both *B. pertussis* and *B. holmesii* [15]. Thus, IS*481*+ samples were tested for *ptxp,* IS*1002* and hIS*1001* amplification to identify *B. pertussis* (*ptxp* + and/or IS*1002*+) and *B. holmesii (*hIS*1001*+) (see Additional file 1). Primers and probes were published elsewhere and are listed in Additional file 2 [14]. qPCR analyses were carried out using the Light Cycler 96 Instrument (Roche). Sequence of *rnasep* human gene was amplified to ascertain quality of sampling and laboratory procedures. Purified DNA from *Bordetella* reference strains and non-template control samples (PCR-grade water) were included as positive and negative qPCR control samples, respectively, in each qPCR run.

Quality control samples and *B. pertussis*, *B. parapertussis*, and *B. holmesii* positive control samples were kindly given by the National Reference Centre of whooping cough and other *Bordetella* diseases, Institut Pasteur, Paris.

For culture, nasopharyngeal swab was streaked on fresh Bordet Gengou mediums (Difco, USA) containing 10% defibrinated horse blood with and without cephalexin (40 μg/mL) (Sigma Chemical Co., USA). After incubation, suspected colonies were confirmed as *B. pertussis* or *B. parapertussis* using qPCR targeting ptxp and IS*481*, and IS*1001* sequences, as previously described, respectively.

#### Serology analysis for indirect diagnosis

Upon reception by the laboratory, blood was immediately spun, and serum was stored at − 20 °C for later analysis. Quantitative assessment of anti-pertussis toxin (PT) immunoglobulin G (IgG) was performed using a purified PT-containing Enzyme-linked immunosorbent assay (ELISA) kit (EUROIMMUN, Ref. EI 2050-G) [16], and the WHO reference serum available from the National Institute for Biological Standards and Control (NIBSC). Results were given in International Units (IU)/ml. Lower limit of quantitation (LLOQ) was defined as 5 IU/ml.

For indirect diagnosis, a cut-off was set at 100 IU of anti-PT IgG titers to define a *B. pertussis* positive contact, at one-year distance from a planned immunization as recommended by National schedule.

#### Definition of *Bordetella pertussis* case

Full-range cycle threshold (CT) value was considered for IS*481*+ qPCR [17]. Among IS*481*+ sample, *B. pertussis* species was molecularly confirmed with IS*1002*+ and/or *ptxp*+, and/or epidemiologically when *B. pertussis* species was confirmed in at least one contact case by serology or by qPCR (see Additional file 1). *B. pertussis* infection could not be confirmed for all IS*481*+ biological samples, those samples were excluded for analysis.

#### Definition of source of infection

Identification of potential source of infection of index cases was performed based on date of cough onset of symptomatic contact and relative index cases, as reported at inclusion or during clinical follow-up. A contact was classified as primary case if he/she started coughing ≥7 days before the index cough onset, and was coughing for ≥12 days. A contact was classified as secondary case if he/she started coughing 1 to 6 days before, the same day, or after the index cough onset.

### Timeliness of pertussis immunization

Timeliness of primary vaccination schedule in infants was analyzed based on the national recommendations. Definition used was the following: having received the 1st dose at 2 months +/− 14 days, and the 2nd and 3rd doses at 4 to 10 weeks interval from the previous dose [10].

### Statistical analyses

Anthropometric z-scores (wasting as weight-for-height, stunting as height-for-age) were calculated and interpreted using the 2006 WHO child growth standards [18]. For continuous variables, medians, interquartile ranges (IQR), minimums and maximums were calculated. Comparisons were carried out using a Student test or a non-parametric Wilcoxon test. When necessary, continuous variables were categorized based on median values or values commonly acknowledged in the literature. Spearman’s coefficient was used to assess rank correlation. For categorical variables, averages were calculated, and comparisons were carried out using the two-sided Chi^2^ or Fisher’s Exact test. To control the confounding factors and to identify the risk factors associated with pertussis infection, a logistic regression analysis was performed. Hematological test results were considered if performed within 4 days from inclusion date. Denominators may differ across analyses due to missing information in questionnaires. Differences were considered significant at *p* < 0.05.

## Results

### Study population

**Fig. 1:**
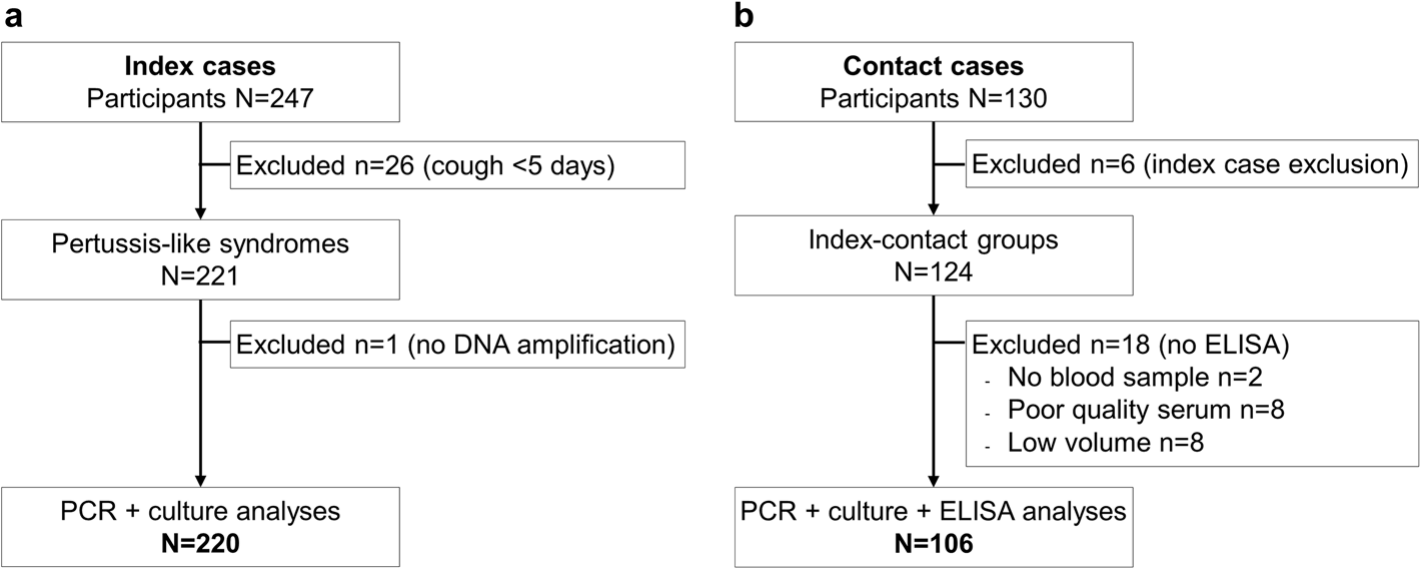
Flow chart of index (**a**) and contact (**b**) cases included in the analysis

A total of 247 infants were enrolled over a period of 31 months (Fig. 1a). Twenty-six infants were subsequently excluded due to screening failure and one due to qPCR analysis failure. Biological analyses were performed for the 220 remaining infants. All infants lived in Tehran province.

A total of 130 contacts of qPCR-positive index cases were enrolled (Fig. 1b). Six contacts were excluded due to relative index case exclusion, 18 were excluded due to the absence, poor quality (hemolysis) or low amount of serum samples. Biological analyses were performed using information from 106 individuals who were contact cases of 39 qPCR-positive index cases.

### Index cases

#### Pertussis diagnosis

Final diagnosis was obtained for 199/220 infants. For 21/220 infants exhibiting IS*481* CT value > 35, *B. pertussis* infection could not be confirmed, these infants were excluded from analysis. A total of 40/199 (20.1%) infants were confirmed to have a *B. pertussis* infection (Table 1). Similar positivity rates were observed in sites 1 and 2, with 18.1 and 21.6% of *B. pertussis* positive infants, respectively (*p* = 0.55). Only 4/40 *B. pertussis* were isolated, including 1 co-infected with *B. parapertussis*. No *B. holmesii* was either detected by qPCR or isolated among those cases.

**Table 1:** Index case description

	Total	***B. pertussis*** cases	Negative cases	
	***N*** = 199	***N*** = 40 (20.1%)	***N*** = 159 (79.9%)	
	N (%)	n (%)	n (%)	*P value*
***Site***				
1	83 (41.7)	15 (37.5)	68 (42.8)	*0.546*
2	116 (58.3)	25 (62.5)	91 (57.2)	
***Gender***				
Boys	115 (57.8)	23 (60.0)	91 (57.2)	*0.751*
Girls	84 (42.8)	16 (40.0)	68 (42.8)	
***Age (months)***				
Median (IQR)	2.0 (1.3–2.8)	2.2 (1.4–2.9)	1.9 (1.2–2.8)	*0.227*
< 3	157 (79.9)	31 (77.5)	126 (79.3)	*0.809*
≥ 3	42 (21.1)	9 (22.5)	33 (20.7)	
***Pertussis immunization***				
Unknown	2 (0.0)	0 (0)	2 (0.0)	
Non-vaccinated	124 (62.3)	20 (50.0)	104 (65.4)	*–*
Age > 75 days	4 (3.2)	1 (5.3)	3 (2.9)	*0.624*
Vaccinated	73 (36.7)	20 (50.0)	53 (33.3)	*–*
Booklet - 1 dose	58 (29.4)	17 (42.5)	41 (26.1)	*–*
Booklet - 2 doses	12 (6.1)	1 (2.5)	11 (7.0)	*–*
Booklet - 3 doses	2 (1.0)	1 (2.5)	1 (0.6)	*–*
Declarative	1 (0.5)	1 (2.5)	0 (0)	*–*
>> > Timeliness	71			
Dose 1	68 (95.8)	19 (100)	49 (94.2)	*0.285*
Dose 2	14 (100)	2 (100)	12 (100)	*–*
Dose 3	2 (100)	1 (100)	1 (100)	*–*

#### Pertussis immunization history

Median age of infants was 2 months and ranged from 8 days to 6 months (Table 1). Distribution of age was similar among positive and negative infants with 79.9% who were less than 3 months old (Table 1). As expected, there was a strong positive correlation between the number of vaccine doses an infant had received and his/her age (*r*_*s*_ = 0.8091; *p* < 0,001). Most infants were unvaccinated (124/199; 62.3%), including 4 who were 2.5 to 5.2 months old and thus already above the higher limit of age in Iran for dose 1 injection (defined as 75 days). There was 73/199 (36.7%) vaccinated infants, mostly with one dose only (58/73). Compliance with recommended age for dose 1 injection was 95.8, and 100% of infants who had more than one dose complied with subsequent dose intervals.

Among *B. pertussis* positive index cases, 1/40 infants was fully and timely vaccinated as recommended by National schedule; third vaccine injection was achieved 12 days before nasopharyngeal sampling.

#### Clinical assessment

Respiratory symptoms and biological information were analyzed and compared among confirmed *B. pertussis* positive and negative index cases (Table 2). Median duration of cough at the time of enrollment was 8 days with 82.9% of infants reporting less than 14 days of cough. Duration was significantly longer among *B. pertussis* positive as compared to negative index cases. Coughing spells (81.9%), good condition between cough (70.6%), cyanosis (65.3%), and nocturnal cough (52.3%) were those most frequently observed. None of the symptoms was significantly associated to biological diagnosis. Abnormal pulmonary auscultation and temperature were rarely observed (13.5 and 4.5%, respectively). Hematological analysis evidenced that leukocyte and lymphocyte counts were significantly higher among positive children as compared to those tested negative (*p* < 0.001 for both), and absolute hyper lymphocytosis was observed in 54.3% of the *B. pertussis* positive infants and 18.8% of the negative infants. Prematurity, low birth weight and scores associated to malnutrition were not associated to pertussis diagnosis. An antibiotic therapy had already been given to 63.8% of the infants before enrollment in the study. Macrolide treatment was used by 43.6% of infants, including by 16/40 of those tested positive for pertussis. Most of the infant enrolled were hospitalized (95.5%).

**Table 2:** Clinical description and antibiotic therapy of *B. pertussis* positive and negative index cases

		N available	Total	***B. pertussis*** cases	Negative cases	*P value*^*a*^
			*N* = 199	*N* = 40	*N* = 159	
***Cough duration at inclusion (days)***						
	median (IQR)	199	8 (6-11)	10 (7.5-19.5)	8 (6-10)	***0.004***
≥ 14 days	n (%)	199	34 (17.1)	15 (37.5)	19 (12.0)	***<0.001***
***Pertussis-like symptoms***						
Nocturnal cough	n (%)	199	104 (52.3)	22 (55.0)	82 (51.6)	*0.698*
Coughing spells	n (%)	199	163 (81.9)	34 (85.0)	129 (81.1)	*0.570*
Apnea	n (%)	199	31 (15.6)	9 (22.5)	22 (13.8)	*0.177*
Cyanosis	n (%)	199	130 (65.3)	31 (77.5)	99 (62.3)	*0.070*
Inspiratory whoop	n (%)	199	32 (16.1)	9 (22.5)	23 (14.5)	*0.216*
Difficult breathing in	n (%)	199	83 (41.7)	17 (42.5)	66 (41.5)	*0.910*
Vomiting	n (%)	199	49 (24.6)	10 (25.0)	39 (24.5)	*0.951*
Post tussif vomiting	n (%)	199	57 (28.6)	16 (40.0)	41 (25.8)	*0.076*
Good conditions btw cough	n (%)	197	139 (70.6)	24 (60.0)	115 (73.3)	*0.101*
Abnormal pulm auscultation	n (%)	193	26 (13.5)	8 (21.1)	18 (11.6)	*0.127*
*>> sign of upper infection*	n (%)	*23*	*14 (60.9)*	*4 (50.0)*	*10 (66.7)*	*0.435*
*>> sign of lower infection*	n (%)		*9 (39.1)*	*4 (50.0)*	*5 (33.3)*	
***Temperature***						
≤ 37.5	n (%)	199	190 (95.5)	38 (95.0)	152 (95.6)	*0.871*
37.5-38.5	n (%)		9 (4.5)	2 (5.0)	7 (4.4)	
***Biological data***						
Leukocytes (/mm^3^)	median (IQR)	191	10600 (8200-13200)	12900 (10100-17300)	9950 (7800-12400)	***<0.001***
Lymphocytes (/mm^3^)	median (IQR)	179	6640 (5150-9265)	10080 (6020-14245)	6438 (5073-8421)	***<0.001***
Hyperlymphocytosis (> 9000/μl)	n (%)	179	46 (25.7)	19 (54.3)	27 (18.8)	***<0.001***
Neutrophiles (/mm^3^)	median (IQR)	179	2772 (1900-4350)	3290 (1800-4862)	2662 (1944-4047)	*0.495*
Platelets (x10^3^/mm^3^)	median (IQR)	193	407 (304-506)	474 (320-605)	392 (297-485)	***0.040***
Hemoglobin (g/dl)	median (IQR)	195	10.5 (9.8-11.5)	11 (9.9-11.6)	10.5 (9.7-11.5)	*0.415*
Mean corpuscular volume (MCV) (μ^3^)	median (IQR)	194	87 (82-93)	87 (81-93)	87 (82.5-93)	*0.567*
SpO2	median (IQR)	196	95 (95-96)	95 (95-96)	95 (95-96)	*0.856*
***Birth and weight***						
Premature birth (< 37 weeks)	n (%)	197	22 (11.2)	2 (5.0)	20 (12.7)	*0.165*
Low birth weight (< 2.5 kg)	n (%)	198	22 (11.1)	2 (5.0)	20 (12.7)	*0.169*
Wasting	n (%)	188	54 (28.7)	13 (34.2)	41 (27.3)	*0.403*
Stunting	n (%)	196	23 (11.7)	6 (15.0)	17 (10.9)	*0.472*
***Antibiotic therapy before enrollment***						
Yes	n (%)	188	120 (63.8)	24 (63.2)	96 (64.0)	*0.923*
Macrolides	n (%)		82 (43.6)	16 (42.1)	66 (44.0)	*0.833*
>> for > 48h before sampling	n (%)	82	37 (45.1)	10 (62.5)	27 (40.9)	*0.119*
***Last clinical evaluation***						
Time after inclusion (days)	median (IQR)	195	14 (13-14)	14 (13-14)	14 (13-14)	*0.933*
Full recovery	n (%)	195	164 (84.1)	27 (67.5)	137 (88.4)	***0.002***
Healing with persistent cough	n (%)	195	27 (13.9)	10 (25.0)	17 (11.0)	*-*
No improvement	n (%)	195	2 (1.0)	2 (5.0)	0 (0-)	*-*
Death	n (%)	195	2 (1.0)	1 (2.5)	1 (0.7)	*-*
***Hospitalization***						
Yes	n (%)	199	192 (96.5)	39 (97.5)	153 (96.2)	*0.696*

^a^*P* values in bold indicate *p* < 0.05

Two deaths occurred during the study. One of these two infants was an unvaccinated 1-month-old infant who was confirmed positive for pertussis infection and had hyperlymphocytosis. The second infant who was diagnosed negative for pertussis had no hyper lymphocytosis, no information was reported regarding possible diagnosis. Both cases had temperature 38 °C and 38.5 °C, respectively, and had coughed for 7 days at the moment of death.

### Contact cases

A median of 3 contact cases were enrolled per index case, and numbers ranged from 1 to 5. There were fathers and mothers (71/106), siblings (22/106), grandfathers and grandmothers (8/106), uncles and aunts (3/106) and nurses (2/106).

#### Pertussis diagnosis

A total 104/106 contact cases had a final diagnosis. Pertussis infection was confirmed for 53/104 (51.0%) contact cases by qPCR (36/104), serology (9/104) or both (8/104) (Table 3). Negative biological confirmation was obtained for 51/104 (49.0%) contacts. One *B. pertussis* was isolated by culture, no *B. holmesii* and no *B. parapertussis* were detected either by qPCR or culture among contacts.

**Table 3:** Pertussis positive contact case description

		Total	Asymptomatic	Symptomatic at inclusion	Symptomatic during study
		***N*** = 53	***N*** = 15 (28.3%)	***N*** = 36 (67.9%)	***N*** = 2 (3.8%)
***Age***					
	median (IQR)	31 (18-36)	31 (24-35)	30.5 (10.5-36)	46.5
	min-max	2-59	12-57	2-59	41-52
***Relationship with index***					
Mothers	n (%)	18 (34.0)	6 (40.0)	12 (33.4)	0 (0)
Fathers	n (%)	17 (32.1)	7 (46.7)	9 (25.0)	1 (50.0)
Siblings (<10 yo^a^)	n (%)	9 (17.0)	0 (0)	9 (25.0)	0 (0)
Siblings (≥10 yo^a^)	n (%)	4 (7.6)	1 (6.7)	3 (8.3)	0 (0)
Gdfathers/Gdmothers^b^	n (%)	3 (5.7)	1 (6.7)	1 (2.8)	1 (50.0)
Nurses	n (%)	2 (3.8)	0 (0)	2 (5.6)	0 (0)
***Pertussis diagnosis***					
PCR+ only	n (%)	36 (67.9)	12 (80.0)	22 (61.1)	2 (100)
Serology+ only	n (%)	9 (17.0)	2 (13.3)	7 (19.4)	0 (0)
PCR+ and serology+	n (%)	8 (15.1)	1 (6.7)	7 (19.4)	0 (0)
***Pertussis vaccination history***					
Siblings <10 yo*	n	9	0	9	0
Non-vaccinated	n (%)	2 (22.2)	-	2 (22.2)	-
Vaccinated	n (%)	7 (77.8)	-	7 (77.8)	-
>> unknown doses	n (%)	3 (42.9)	-	3 (42.9)	-
>> 4 doses	n (%)	4 (57.1)	-	4 (57.1)	-
***Macrolide treatment before inclusion***					
No	n (%)	26 (49.1)	8 (53.3)	17 (42.2)	1 (50.0)
Yes	n (%)	17 (32.1)	2 (13.3)	14 (38.9)	1 (50.0)
Unknown	n (%)	10 (18.9)	5 (33.3)	5 (13.9)	0 (0)

^a^*yo* years old, ^b^*gdfathers/gdmothers* grandfathers / grandmothers

*B. pertussis* positive contact cases were mostly adults (75.5%) with median age of 31 years, ranging 2 to 59 years old (Table 3). They included mothers (34.0%), fathers (32.1%), < 10 years old siblings (17.0%), ≥ 10 years old siblings (7.6%), grandfathers and grandmothers (5.7%) and nurses (3.8%) (Table 3).

#### Pertussis vaccination history

Among siblings aged < 10 years who were tested positive (9/53), 100% were confirmed *B. pertussis* positive by qPCR, regardless of their serology result, and had cough. No information was obtained regarding the date of their last pertussis vaccination. However, 2/9 aged 8 and 9 years reported to be not vaccinated, and 7/9 were vaccinated with 4 doses but did not receive the 5th dose yet (they were 2, 6, 7 and 8 years old) or with unknown number of dose (they were 3, 3 and 5 years old).

#### Clinical assessment

Contact cases did not report symptoms (15/53), were symptomatic with cough onset reported during the study (2/53), or symptomatic with reported cough at inclusion (36/53).

Among the 15 asymptomatic contact cases, *B. pertussis* was detected by qPCR (12/15), by serology (2/15), or by both assays (1/15) (Table 3). Regarding the use of macrolide antibiotics, 2/15 had a treatment (azithromycin) before inclusion (4 and 9 days), which may have interfered with cough onset. For 5/15 contacts the use of any macrolide antibiotics was not known while 8/15 did not receive any treatment. Asymptomatic contact cases included 13/15 fathers and mothers, 1/15 grandmother and 1/15 twelve-years-old sister.

Contacts who reported cough onset during the study (2/53) were confirmed positive by qPCR and were negative by serology.

There were 36/53 contact cases presenting cough at inclusion, they included all the pertussis positive contact aged < 10 years. Nocturnal cough (42.9%), coughing spells (45.7%) and good condition between cough (57.1%) were mainly reported (Table 4).

**Table 4:** Clinical assessment of symptomatic contact cases at inclusion

***Symptoms reported at inclusion***		***N*** **=** **36**
Duration of cough (days)	median (IQR)	17.5 (9–32)
≥ 7	n (%)	31 (86.1)
≥ 14	n (%)	20 (55.6)
Nocturnal cough ^a^	n (%)	15 (42.6)
Coughing spells ^a^	n (%)	16 (45.7)
Apnea ^a^	n (%)	0 (0)
Cyanosis ^a^	n (%)	0 (0)
Inspiratory whoop ^a^	n (%)	1 (2.9)
Difficult breathing in ^a^	n (%)	3 (8.6)
Vomiting ^a^	n (%)	0 (0)
Post tussif vomiting ^a^	n (%)	1 (2.9)
Good conditions btw cough ^a^	n (%)	20 (57.1)

^a^ *n* = 1 with no information available

### Source of infection

**Fig. 2:**
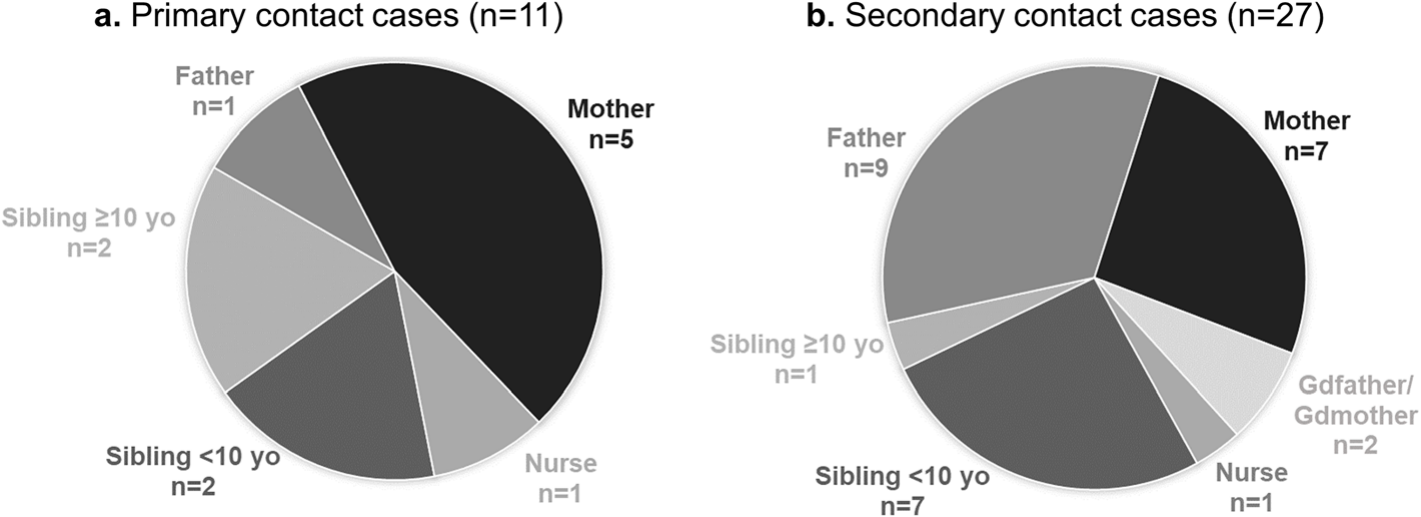
Distribution of primary (**a**) and secondary (**b**) cases with respect to the index case among positive contacts. yo, years old

Symptomatic contact cases (38/53) were further classified as primary or secondary cases based on the time of cough onset. There were 11/53 (20.8%) contact cases identified as potential primary cases, they were all coughing for more than 20 days at index cough onset (Fig. 2a). A single primary case was identified for five index cases; there were a nurse (*n* = 1), a 12-year-old sibling who declared to be vaccinated (*n* = 1), and the mother (*n* = 3). For two index cases, several potential source of infection were identified and included the mother, the father and two non-vaccinated siblings aged 8 and 9 years, and the mother and a 14-year-old brother who had received four doses of pertussis vaccine during infancy only.

Twenty-seven symptomatic (50.9%) contact cases contracted pertussis infection along or after the infants (Fig. 2b).

## Discussion

This prospective hospital-based study shows that *B. pertussis* infection was confirmed in 20.1% (40/199) of the infants presenting ≥5 days of cough associated to one pertussis-like symptom enrolled in two main hospitals in Tehran, and highlights the high circulation of the pathogen among their contacts aged 2 to 59 years old.

In infants, clinical and biological evaluation evidenced that higher number of leukocytes, lymphocytes, platelets and cough duration were significantly associated to pertussis infection. While the relationship between platelets and pertussis is controversial, the association of white blood cell number and cough duration with pertussis was previously described in Iran and in other settings, including in HIC [19, 20]. Of note, we observed that 62.5% of the confirmed positive infants coughed less than 14 days when seeking diagnosis. However, additional symptoms typically associated to pertussis (i.e. nocturnal or coughing spells, apnea, cyanosis, inspiratory whoop, difficulty breathing in, post-tussif vomiting) were not found to be more frequent in infants with pertussis in this study. As expected, absence of fever and normal pulmonary auscultation were mainly reported among pertussis positive infants, nevertheless it was not specifically associated to positivity either. A recent study in Italy developed an algorithm based on clinical symptoms to predict pertussis infection, however, although it may be a good predictor when using local data, our results highlighted that qPCR testing is indispensable for pertussis diagnosis [21].

A National Pertussis Reference laboratory is established in Tehran, but only a few samples collected from different areas of the country are sent to that laboratory for biological testing [8]. Given the difficulty of clinically diagnosing pertussis, biological diagnosis should be more widely used for National surveillance. It is particularly important as its sensitivity has greatly improved with the use of qPCR targeting the IS*481* sequence that is present at a high copy number within *B. pertussis’* genome. In fact, while only five samples were successfully cultured during this study, 84 cases were confirmed as pertussis positive by qPCR. The use of macrolide antibiotics a few days before sampling, the presence of Amies buffer as sample transport media, or the delay for culturing sample that was sometimes up to 24 h may have impaired culture. Nevertheless, when samples contain low quantity of target, IS*481* amplification may be low (CT > 35) and sample might be interpreted as negative as fixed positive diagnosis threshold is commonly defined as CT ≤ 35. However, Gill and colleagues recently showed how the burden of pertussis may be substantially underestimated when restricting diagnostic criteria to IS*481* CT ≤ 35 [17]. Herein, correlating with Gill et al., we identified 6 index cases having IS*481* CT > 35 but for whom up to four of the respective contact cases exhibited *B. pertussis* confirmed qPCR and/or serology. However, in absence of epidemiological confirmation or repeated nasopharyngeal sampling over time, uncertainty remains on the positivity of the sample [17]. In addition, for research purposes, amplification of specific sequences (such as *ptxp*, IS*1002*, hIS*1001*) are needed to confirm *B. pertussis* species among IS*481*+ samples, sequence also present in *B. holmesii* genome, but not always possible as those sequences are present at a single or low copy number within bacterial genome [22].

In this study, although all infants aged 0–6 months old exhibiting pertussis-like syndrome were considered, enrolled infants were mostly less than 3 months of age (80%) and were either non-vaccinated or had received a single dose (> 90%). To prevent severe pertussis cases in infants, WHO recommended that every country should seek to achieve early and timely vaccination initiated at 6 weeks of age and no later than 8 weeks [3]. Only 4 infants were late in starting the vaccination program among the 199 participating infants, and > 90% of those infants who were vaccinated complied with the national vaccination schedule in this study. Compliance with vaccination schedule was also found to be very good among vaccinated children 3–15 years old in Tehran, confirming our data [10]. Timeliness may nevertheless vary throughout the country as high incidence of delayed vaccination was reported in the outskirt of Iran’s big cities [23].

Due to the arrangement of family unit in Tehran, only a few numbers of contact cases were included and several index case had no or few contacts. Among the 39 infants for whom contacts were tested, 33 had at least one contact with a confirmed *B. pertussis* infection. Overall, more than half of the contact cases were tested positive for pertussis infection. All age groups were concerned and median age was 31 years old. Parents were more frequently tested as positive contact (66.0%), followed by siblings (24.5%). This relationship with infant and age distribution of primary case contacts was recently shown to be similar in HIC where aPVs are used [24]. The important rate of pertussis infection among contact cases observed herein highlights the underreporting among older children and adults and are in line with studies evidencing substantial circulation of pertussis among Iranian children and adolescents [10, 25]. In addition, although all positive children presented symptoms, more than 30% of older individuals did not recall having or did not have cough at any time during the study. When cough was reported, there was no additional symptom, as often reported in pertussis-infected adults [26, 27]. In this study 11 possible primary cases were identified who were contacts of 7/39 (17.9%) positive infants. More than 90% were household contacts (mothers, siblings and father represented 5/11, 4/11, 1/11, respectively). The Wiley et al.’s systematic review demonstrated that, when identified, the source of infection in infants aged < 6 months in HIC were household contacts in 74–96% of the cases, parents being identified more often, which is in agreement with our findings [28]. Similar observations were found in Brazil [29].

Identification of primary cases was limited. This is due to a lack of awareness of the disease among the general population that impairs identification and report of pertussis symptoms, and to possible subclinical infections. In addition, no symptom typically associated to pertussis, except duration of cough, was found to be associated to a positive molecular diagnosis result of pertussis in infants. This could be related to a lack of power of our study.

In Iran, the implementation of adolescent booster vaccine, which would contribute to increasing herd immunity, was proposed and expected to be cost-effective [30, 31]. Another complementary strategy would be the introduction of maternal immunization, found to be effective around the world including in LMIC, which would contribute to reducing the rate of severe and fatal cases among the newborns [3, 32–35].

## Conclusions

This study evidences substantial prevalence of *B. pertussis* among infants exhibiting cough and among their close contacts. It also shows that biological testing is crucial for diagnosis in infants for whom clinical symptoms are poorly specific, and in contacts who are poorly symptomatic and/or not aware of pertussis symptoms. Sensitive and specific diagnosis tool such as qPCR should be widely used for surveillance.

## Supplementary Information

**Additional file 1: Figure S1.** Decision tree flowchart for *Bordetella* species identification for biological diagnosis using qPCR assays.

**Additional file 2: Table S1.** Primer and probe sequences for TaqMan technology-based real time PCR assays.

## Data Availability

The datasets used and/or analyzed during the current study are available as de-identified, from the corresponding author on reasonable request.

## Abbreviations

LMIC: Low and middle-income countries
wPV: Whole-cell vaccine
HIC: High-income countries
aPV: Acellular vaccine
qPCR: Real-time quantitative polymerase chain reaction
PT: Pertussis toxin
IgG: Immunoglobulin G
ELISA: Enzyme-linked immunosorbent assay
WHO: World Health Organization
NIBSC: National Institute for Biological Standards and Control
IU: International units
LLOQ: Lower limit of quantitation
CT: Cycle threshold
IQR: Interquartile range
yo: Years old
gdfathers/gdmothers: Grandfathers / grandmothers
