# Supplementary figures and images for: Hospital-based prospective study of pertussis in infants and close contacts in Tehran, Iran
Source: BMC Infect Dis. 2021 Jun 18;21:586. doi: 10.1186/s12879-021-06266-6 (PMC8212501; doi:10.1186/s12879-021-06266-6)

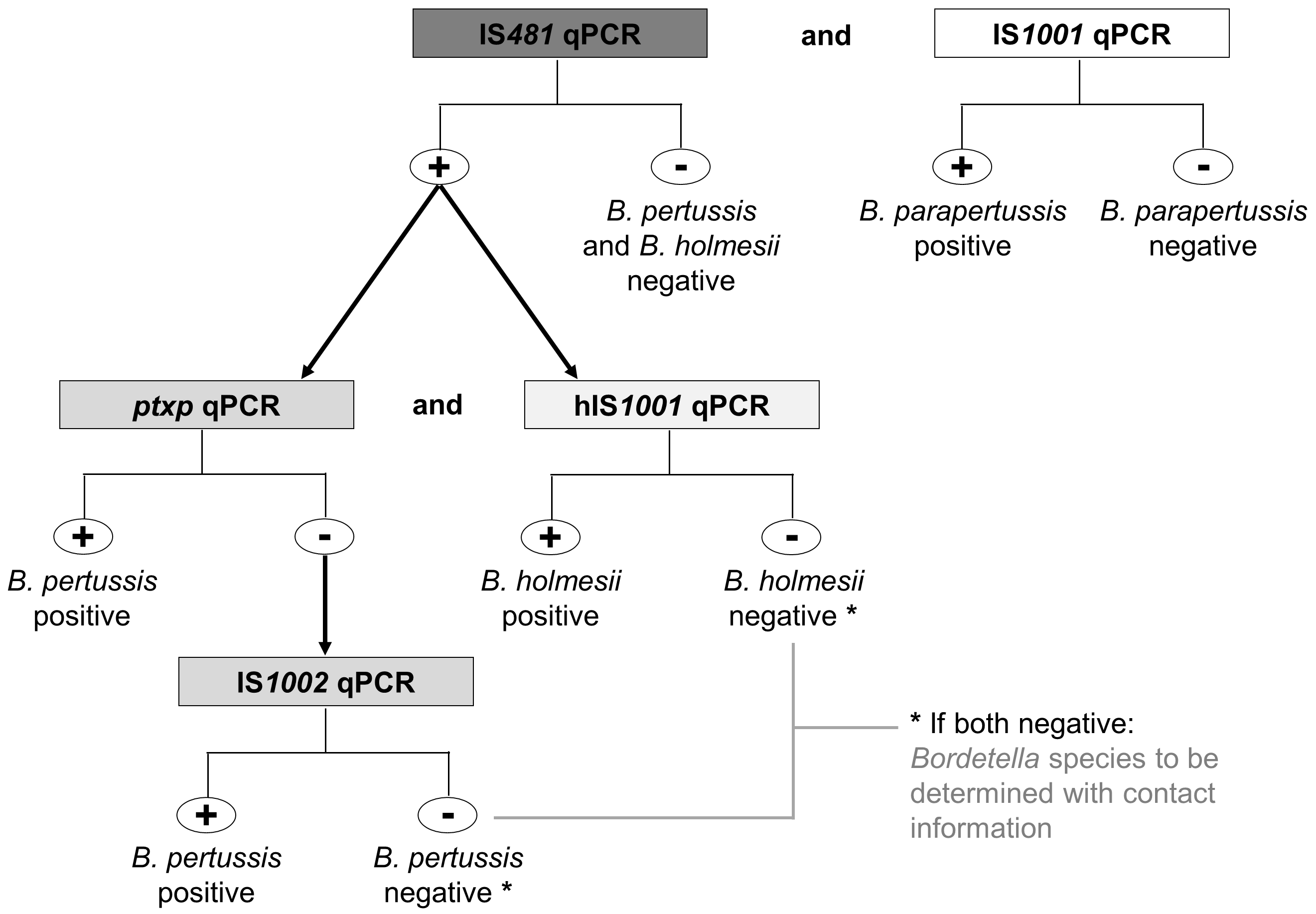

Supplement: Supplementary file 1 — Additional file 1: Figure S1. Decision tree flowchart for Bordetella species identification for biological diagnosis using qPCR assays. [file 12879_2021_6266_MOESM1_ESM.tif]
